# Classification of COVID-19 in intensive care patients

**DOI:** 10.1186/s13054-020-03127-7

**Published:** 2020-07-09

**Authors:** Xiaofan Lu, Yang Wang, Taige Chen, Jun Wang, Fangrong Yan

**Affiliations:** 1 State Key Laboratory of Natural Medicines, Research Center of Biostatistics and Computational Pharmacy, China Pharmaceutical University, Nanjing, 210009 China; 2 Department of Radiology, The Affiliated Nanjing Drum Tower Hospital of Nanjing University Medical School, Nanjing, 210008 China; 3 Medical School of Nanjing University, Nanjing, 210093 China; 4 Department of Intensive Care Medicine, The First Affiliated Hospital of Soochow University, Suzhou, 215006 China

Dear Editor,

Previous studies on coronavirus disease 2019 (COVID-19) mainly described patients’ general information [1]. We aimed to bridge the gap between disease classification and clinical outcome in intensive care patients, which could help in the individual evaluation and provide effective triage for treatment and management.

One hundred fifty-one intensive care patients with complete medical records were obtained from Tongji Hospital in Wuhan, China. Data on the day of admission were collected, including six data categories: demographic information of age and gender, symptoms ([> 10%] fever, fatigue, dry cough, anorexia, myalgia, dyspnea, expectoration, diarrhea), original comorbidities ([> 5%] hypertension, diabetes, cardiovascular disease [CVD], chronic obstructive pulmonary disease [COPD], malignancy), vital signs (respiratory rate, heart rate, blood pressure, SpO_2_, FiO_2_), blood routine tests (count of WBC, lymphocyte, neutrophil, platelet and monocyte, red cell distribution width [RDW]), and inflammatory marker measurements (high-sensitivity C-reactive protein [hs-CRP], interleukin-2 receptor [IL-2R], IL-6, IL-8, IL-10, TNF-α). Blood routine tests were also measured at days 3 and 5 since admission, and adjuvant corticosteroid therapy throughout the disease course was retrieved. Clinical outcome was 28-day mortality after admission. The Ethics Commission of Tongji Hospital approved this study, with a waiver of informed consent. We constructed a fully Bayesian latent variable model for integrative clustering of the six data categories [2]. The appropriate clustering number was determined by minimizing the Bayesian information criterion. Only features with high posterior probability (e.g., 0.8) were kept.

We identified four prognostic types of COVID-19 (Fig. 1). The characteristics of the four types were described below (Table 1). *Type A:* Extremely poor prognosis and elderly enriched; Dry cough, dyspnea, and fatigue were common symptoms; hypertension, diabetes, and CVD were common preexisting medical conditions. Patients presented severe respiratory failure, dramatically elevated counts of WBC and neutrophil, and lymphocyte depletion. Remarkably elevated cytokine occurred, accompanied by later development of ARDS and multiple organ failure. *Type B:* Poor prognosis and elderly enriched; dyspnea and cough with expectoration were common symptoms, accompanied by diarrhea and anorexia. Unfavorable respiratory condition and decreased lymphocyte count could be observed. Patients presented an imminent elevation of cytokine and a high risk of developing ARDS and multiple organ failure later after treatment. *Type C:* Intermediate prognosis; symptoms of dry cough and fatigue, and original comorbidity of hypertension were common. The respiratory condition was normal, and most laboratory tests were within normal or moderately elevated. *Type D:* Favorable prognosis and middle age enriched; primary symptom was cough with expectoration. Patients had stable breathing and most laboratory tests were in a normal range or slightly elevated.

**Fig. 1:**
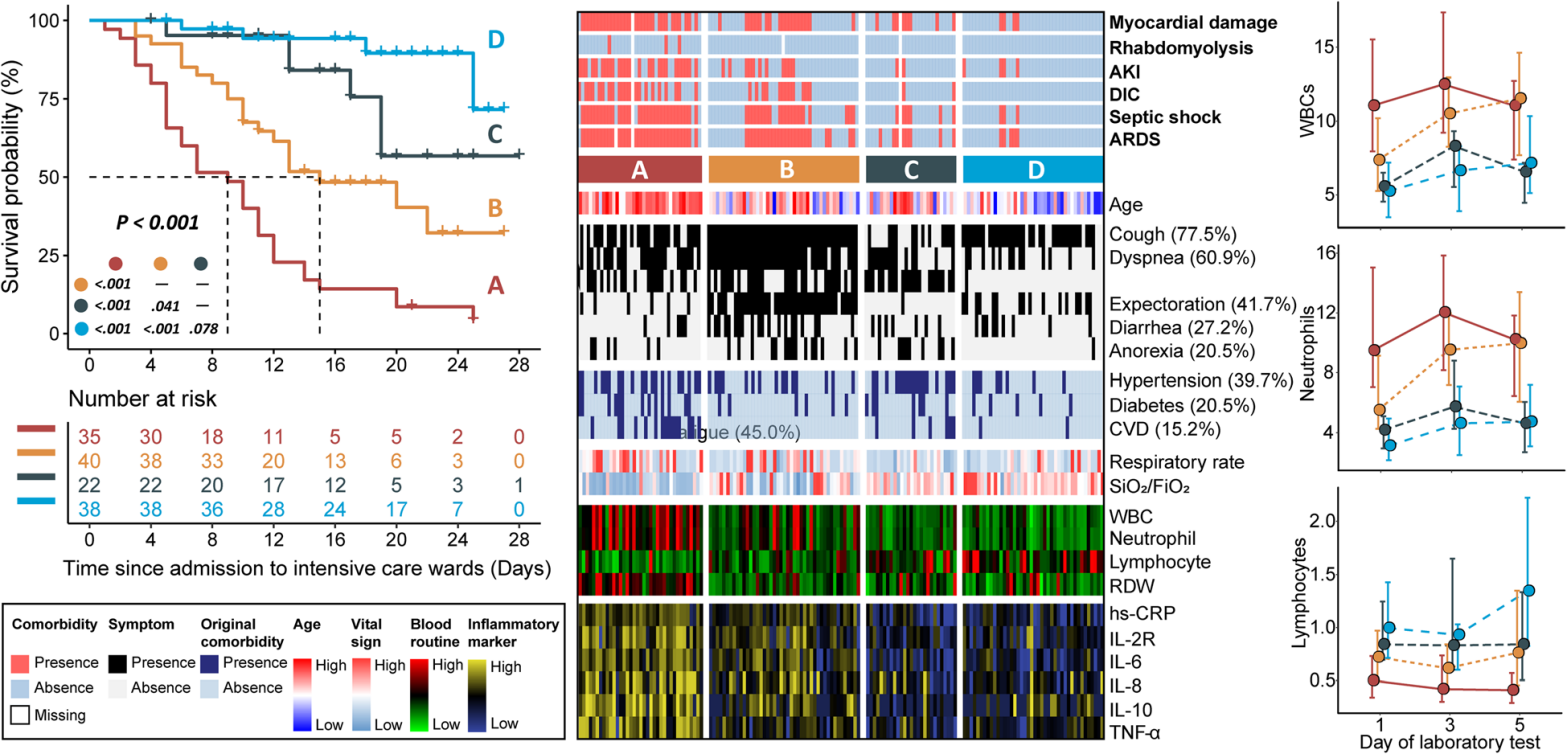
Clinical landscape of four prognostic types of COVID-19 in intensive care patients. Kaplan-Meier survival curves (left panel) showing differential survival rates; comprehensive heatmap (middle panel) delineating clinical landscape of different types of COVID-19, with legend positioning in the left bottom panel; time line charts (right panel) profiling the dynamic measurement (median [IQR], × 10^9^/L) of blood routine tests at days 1, 3, and 5 since admission among four prognostic types. Survival was analyzed with log-rank test and pair-wise comparison was adjusted by Benjamini-Hochberg method. Labels of “high” and “low” were based on data interval instead of clinical reference values. AKI: acute kidney injury; DIC: disseminated intravascular coagulation; ARDS: acute respiratory distress syndrome; CVD: cardiovascular disease; SpO_2_: peripheral oxygen saturation; FiO_2_: fraction of inspired oxygen; WBC: white blood cell; RDW: red cell distribution width

**Table 1:** Presenting characteristics of four types of COVID-19 in intensive care patients (*n* = 151)

	A (***n*** = 37)	B (***n*** = 45)	C (***n*** = 27)	D (***n*** = 42)
Age, years	77 (70–81)	62 (52–70)	65 (51–74)	53 (43–58)
Signs and symptoms				
Cough	26 (70)	45 (100)	12 (44)	34 (81)
Dyspnea	24 (65)	41 (91)	14 (52)	13 (31)
Fatigue	19 (51)	27 (60)	21 (78)	1 (2)
Expectoration	6 (16)	40 (89)	1 (4)	16 (38)
Diarrhea	5 (14)	20 (44)	7 (26)	9 (21)
Anorexia	6 (16)	15 (33)	9 (33)	1 (2)
Original comorbidities				
Hypertension	20 (54)	15 (33)	17 (63)	8 (19)
Diabetes	13 (35)	6 (13)	7 (26)	5 (12)
CVD	15 (41)	0	6 (22)	2 (5)
Vital signs				
Respiratory rate, rpm	25 (20–32)	22 (20–26)	20 (20–23)	21 (20–25)
SpO_2_/FiO_2_	99 (90–158)	222 (100–294)	297 (237–336)	298 (248–345)
Laboratory findings				
*Routine blood test*				
WBCs, × 10^9^/L				
Day 1	11.1 (8.0–15.5)	7.4 (5.3–10.2)	5.6 (4.5–6.5)	5.2 (3.5–7.2)
Day 3	12.5 (9.2–17.4)	10.5 (8.3–12.9)	8.3 (5.5–9.3)	6.6 (3.9–8.3)
Day 5	11.1 (7.4–12.7)	11.5 (7.7–14.6)	6.6 (4.5–7.2)	7.2 (5.1–10.3)
Absolute lymphocytes, × 10^9^/L				
Day 1	0.5 (0.3–0.7)	0.7 (0.5–1.0)	0.8 (0.7–1.2)	1.0 (0.7–1.4)
Day 3	0.4 (0.3–0.7)	0.6 (0.4–0.8)	0.8 (0.6–1.7)	0.9 (0.6–1.0)
Day 5	0.4 (0.3–0.6)	0.8 (0.5–1.3)	0.8 (0.5–1.3)	1.3 (0.8–2.2)
Absolute neutrophils, ×10^9^/L				
Day 1	9.5 (7.1–15.0)	5.5 (4.3–9.1)	4.2 (3.0–5.1)	3.1 (2.2–5.0)
Day 3	12.0 (8.2–15.8)	9.5 (7.2–12.0)	5.7 (4.3–8.8)	4.6 (2.5–7.1)
Day 5	10.2 (6.5–11.8)	10.0 (6.1–13.4)	4.6 (2.7–6.1)	4.7 (3.1–7.2)
RDW-CV	13.4 (12.8–14.1)	12.2 (11.9–12.8)	12.6 (11.8–13.0)	12.2 (11.8–12.7)
*Inflammatory marker*				
hs-CRP, mg/L	126 (76–190)	80 (42–109)	19 (5–49)	28 (10–70)
IL-2R, U/ml	1341 (940–1809)	1038 (678–1185)	701 (430–813)	685 (439–928)
IL-6, pg/ml	68 (37–137)	43 (21–79)	10 (2–20)	14 (5–30)
IL-8, pg/ml	42 (21–95)	21 (13–40)	12 (7–24)	15 (10–21)
IL-10, pg/ml	15 (9–24)	7 (3–11)	3 (3–6)	3 (3–9)
TNF-α, pg/ml	15 (10–23)	9 (8–11)	7 (5–9)	8 (7–10)
Corticosteroid therapy	28 (76)	38 (84)	13 (48)	27 (64)

Continuous variables were described as median (IQR) while categorical variables were expressed as frequencies (%). All records were measured at admission to intensive care wards unless otherwise indicated. Multiple group comparison was done with Kruskal–Wallis test; proportions for categorical variables were compared using Fisher’s exact test. All calculated *P* values were less than or equal to 0.001 except for respiratory rate (*P* = 0.004), absolute lymphocytes at day 3 since admission (*P* = 0.013), and corticosteroid therapy (*P* = 0.013)

*Abbreviations*: *CVD* cardiovascular disease, *rpm* breaths per minute, *SpO*_*2*_ peripheral oxygen saturation, *FiO*_*2*_ fraction of inspired oxygen, *WBC* white blood cell, *RDW* red cell distribution width

This report, to our knowledge, is the first attempt of dealing with the classification of COVID-19 in intensive care patients. The four prognostic types present a stepwise distribution in age, respiratory condition, and inflammatory markers, suggesting their prognostic efficacy. The specificity of symptoms does not appear to be strong, but gastrointestinal response (e.g., diarrhea) needs vigilance [3]. Unexpectedly, hypertension is more evenly distributed, which contradicts previous study indicating hypertensive with COVID-19 was more likely to be in a high risk of mortality [4]. Notably, types A and B always showed higher content of WBCs and neutrophils, no matter on days 1, 3, or 5 since admission, while types C and D had relatively higher lymphocyte counts compared to other types; such trend seemed not to be affected by corticosteroids even though more patients in types A and B received adjuvant corticosteroids therapy than C and D. Investigations in larger cohorts are required to provide more evidence. The study is limited by ignoring the potential treatment effect. However, such classification could help in better triage, allowing for a more rational allocation of scarce medical resources in a resource-constrained environment.

## Data Availability

Drs. J. Wang had full access to all of the data in the study. After publication, the data will be made available to others on reasonable requests after approval from the author (J.W, dr_wangjun@suda.edu.cn) and Wuhan Tongji Hospital.
